# Increasing labor force participation in older age requires investments in work ability

**DOI:** 10.5271/sjweh.3941

**Published:** 2020-12-16

**Authors:** Mikko Laaksonen

**Affiliations:** Senior Researcher Finnish Center for Pensions, Helsinki, Finland. [Email: Mikko.Laaksonen@;etk.fi]

For well over 100 years, life expectancy in developed societies has increased by 2.5 years per decade (1). At first, most of the increase was due to decreased death rates at younger ages, but since the 1950s, the main reason has been better survival after age 65. Factors contributing to our increasing lifespan include better living conditions, healthier lifestyles, advances in healthcare and a shift from heavy jobs to non-manual occupations.

Coupled with a long-term fall in fertility rates, the consequence of this increasing length of life has been a growing number and share of the elderly population. Currently, almost one fifth of the population in the European Union (EU) has reached the age of 65, and this share is projected to increase during the next decades. While the increase in life expectancy is a great accomplishment, the growing share of older people also poses a range of economic and social challenges. By 2050, nearly 30% of the EU population will be older than 65, and the old-age dependency ratio will exceed 50% (2). The increasing number of older people relative to the working-age population will exert pressures on economic growth, increase age-related social costs and endanger the sustainability of government finances.

This development has raised the topic of extending working lives high on the political agenda. To counter the economic costs of the growing elderly population, reforms to increase the statutory retirement age, cut the routes to early retirement and increase labor force participation among older workers have been put forward in most European countries (3). This has also led to positive developments, as the average age of transition into retirement has increased in many countries, and the employment rate in older ages has improved (4).

However, a large proportion of people are unable to continue in paid employment until the increasing statutory retirement age. Poor health and work ability are major reasons for early exit from the labor market. Disability retirement is one of the most common pathways for premature exit from working life, but poor health is also associated with exit from work through unemployment (5). Unemployment and poor health are interrelated in complex ways: poor health may increase the risk of unemployment and unemployment, in turn, may increase health problems. Long term, they may form a self-reinforcing cyclical relationship. Unemployment is also associated with an increased risk of disability retirement (6, 7).

Working life expectancy is a measure that summarizes the effect of various, often competing, exit routes on the length of working lives (8). This measure indicates how much of the remaining lifetime will be spent in employment or in labor market activity. It also takes into account the timing of labor market exit so that earlier withdrawal gets more weight. Studies have shown that working life expectancy is shorter in the lower socioeconomic groups (9, 10) and in occupations with high physical demands (11).

In this issue of *The Scandinavian Journal of Work, Environment and Health*, Schram et al (12) analyse the influence of occupational class and physical workload on working life expectancy and lost working years using multistate Cox regression models. In the age range 50–63 years, working life expectancy among manual workers was one year lower than that among upper non-manual employees. Nearly two thirds of this difference was explained by unemployment and one third by disability retirement. Physically demanding work was also related to lost working years, mostly due to ill-health-based exit routes. The effect of physical work on lowered working life expectancy could not be reduced back to occupational class, as it was consistently observed within occupational classes. Manual workers with physically demanding work had the lowest working life expectancy.

Another element in extending working lives is continuing to work after reaching the statutory retirement age. So far, most people have stopped working at that stage and relatively few have continued. However, the proportion of people working beyond the retirement age has been increasing (13). Evidence on factors that affect working beyond the retirement age is more limited than information on premature retirement.

For many, extending working beyond the retirement age is voluntary, but others may be forced to continue working for financial reasons if their retirement income is low. It is known that men, the better educated and those who have recently reached their retirement age are typically more likely to participate in paid work after reaching their retirement age. Own motivation and job satisfaction, as well as many aspects of personal life, such as the partner’s labor market position, are important factors affecting the retirement decision. Many older employees prefer part-time work, and they appreciate possibilities for worktime arrangements and other flexibility (14).

Being in good health is an important precondition for working beyond the retirement age (15, 16). Working conditions also play an important role, and they are obviously related to the previously mentioned motivation, job satisfaction and work ability. Also in this issue, the study by Andersen et al (17) shows that higher physical work demands are associated with a lower likelihood and a good psychosocial work environment with a higher likelihood of working beyond the state retirement age. A good psychosocial work environment is important for both employees in sedentary and physically active work. It has also been shown that employees with higher occupational classes are much more likely to continue working beyond the retirement age compared to those with lower occupational classes. A large proportion of these differences can be explained by physical work demands, work time control, and self-rated work ability (18). Thus, it seems that the factors affecting working beyond the statutory retirement age are, at least in part, similar to factors that relate to early exit.

Despite the generally favorable development in population health, the increasing statutory retirement age means that people approaching the end of their working careers will inevitably encounter chronic conditions (19). Increasing attention is needed to ensure that people are able to continue their working lives despite such conditions. In this respect, work accommodation and vocational rehabilitation activities are of high value. Even though hard physical labor has become less common during recent decades, many jobs still involve physical work demands. Thus, efforts to reduce physical work exposures are needed to prevent early labor market exit among older workers. Such efforts and a continuous development of skills need to be started well in advance. Working lives must also become more flexible for different working abilities and life situations.

An increasing level of education and changes in the occupational structure from physically heavy jobs to service sector occupations may have been partly responsible for the increase in working life expectancy. At the same time, increasing cognitive demands, work-related stress and fast changes in technology pose new challenges. Such psychosocial working conditions may particularly relate to mental work ability (20). If mental health problems are involved, continuing in work may be challenging, regardless of whether the health problems initially originate from the workplace or not. As mental health problems often emerge in young adulthood, they may result in a large number of lost working years and continue to affect one’s life throughout the whole life course (21–23). Therefore, efforts to promote mental health and well-being are increasingly important for maintaining a high labor market participation.

Socioeconomic position is one of the key determinants of extending working lives. So far, those who are better educated and work in higher non-manual occupations seem to be in a better position to continue their working lives. Extending working lives further implies that special attention needs to be paid to occupations where the physical and psychosocial risk factors are common. If all work environments do not allow continued employment, the increase in the length of working lives may be uneven and lead to increasing economic inequalities in older age. Reducing work-related risks and ensuring a healthy and safe work environment for everyone until retirement is also a primary target in tackling health inequalities.

## References

[1] Oeppen J, Vaupel JW (2002). Broken Limits to Life Expectancy. Science.

[2] Eurostat:Ageing Europe - Looking at the lives of older people in the EU (2020).

[3] OECD:Pensions at a Glance 2019 (2019). OECD and G20 Indicators.

[4] Eurostat:Employment - annual statistics //ec.europa.eu/eurostat/statistics-explained/index.php?title=Employment_-_annual_statistics.

[5] van den Berg T, Schuring M, Avendano M, Mackenbach J, Burdorf A (2010). The impact of ill health on exit from paid employment in Europe among older workers. Occup Environ Med.

[6] Stover M, Pape K, Johnsen R, Fleten N, Sund ER, Claussen B, Bjorngaard JH (2012). Unemployment and disability pension - an 18-year follow-up study of a 40-year-old population in a Norwegian county. BMC Public Health.

[7] Bratsberg B, Fevang E, Røed K (2013). Job loss and disability insurance. Labour Econ.

[8] Nurminen MM, Heathcote CR, Davis BA, Puza BD (2005). Working life expectancies:the case of Finland 1980-2006. J R Stat Soc Ser A Stat Soc.

[9] Robroek SJ, Nieboer D, Järvholm B, Burdorf A (2020). Educational differences in duration of working life and loss of paid employment: working life expectancy in The Netherlands. Scand J Work Environ Health.

[10] Leinonen T, Martikainen P, Myrskylä M (2018). Working life and retirement expectancies at age 50 by social class:Period and cohort trends and projections for Finland. J Gerontol B Psychol Sci Soc Sci.

[11] Pedersen J, Schultz BB, Madsen IEH, Solovieva S, Andersen LL (2020). High physical work demands and working life expectancy in Denmark. Occup Environ Med.

[12] Schram JL, Solovieva S, Leinonen T, Viikari-Juntura E, Burdorf A, Robroek SJ (2021). The influence of occupational class and physical workload on working life expectancy among older employees. Scand J Work Environ Health.

[13] Eurofound:Income from work after retirement in the EU (2012).

[14] Scherger S (2015). Paid work beyond pension age. omparative Perspectives.

[15] Wahrendorf M, Akinwale B, Landy R, Matthews K, Blane D (2017). Who in Europe Works beyond the State Pension Age and under which Conditions?Results from SHARE. J Popul Ageing.

[16] Scharn M, van der Beek AJ, Huisman M, de Wind A, Lindeboom M, Elbers CT, Geuskens GA, Boot CR (2017). Predicting working beyond retirement in the Netherlands:an interdisciplinary approach involving occupational epidemiology and economics. Scand J Work Environ Health.

[17] Andersen LL, Thorsen SV, Larsen M, Sundstrup E, Boot CR, Rugulies R (2021). Work factors facilitating working beyond state pension age:Prospective cohort study with register follow-up. Scand J Work Environ Health.

[18] Virtanen M, Oksanen T, Pentti J, Ervasti J, Head J, Stenholm S, Vahtera J, Kivimäki M (2017). Occupational class and working beyond the retirement age:a cohort study. Scand J Work Environ Health.

[19] Parker M, Bucknall M, Jagger C, Wilkie R (2020). Population-based estimates of healthy working life expectancy in England at age 50 years:analysis of data from the English Longitudinal Study of Ageing. Lancet Public Health.

[20] Bonde JPE (2008). Psychosocial factors at work and risk of depression:a systematic review of the epidemiological evidence. Occup Environ Med.

[21] Pedersen J, Thorsen SV, Andersen MF, Hanvold TN, Schlunssen V, Bultmann U (2019). Impact of depressive symptoms on worklife expectancy:a longitudinal study on Danish employees. Occup Environ Med.

[22] Laaksonen M, Rantala J, Järnefelt N, Kannisto J (2018). Educational differences in years of working life lost due to disability retirement. Eur J Public Health.

[23] Knudsen AK, Overland S, Hotopf M, Mykletun A (2012). Lost working years due to mental disorders:an analysis of the Norwegian disability pension registry. PLoS One.

